# Giant intrinsic chiro-optical activity in planar dielectric nanostructures

**DOI:** 10.1038/lsa.2017.158

**Published:** 2018-02-23

**Authors:** Alexander Y Zhu, Wei Ting Chen, Aun Zaidi, Yao-Wei Huang, Mohammadreza Khorasaninejad, Vyshakh Sanjeev, Cheng-Wei Qiu, Federico Capasso

**Affiliations:** 1 John A. Paulson School of Engineering and Applied Science, Harvard University, Cambridge, MA 02138, USA; 2 Department of Electrical and Computer Engineering, National University of Singapore, Singapore 117583, Singapore; 3 University of Waterloo, Waterloo, ON N2L 3G1, Canada

**Keywords:** chiral, dielectric, extrinsic chirality, metasurface, optical activity

## Abstract

The strong optical chirality arising from certain synthetic metamaterials has important and widespread applications in polarization optics, stereochemistry and spintronics. However, these intrinsically chiral metamaterials are restricted to a complicated three-dimensional (3D) geometry, which leads to significant fabrication challenges, particularly at visible wavelengths. Their planar two-dimensional (2D) counterparts are limited by symmetry considerations to operation at oblique angles (extrinsic chirality) and possess significantly weaker chiro-optical responses close to normal incidence. Here, we address the challenge of realizing strong intrinsic chirality from thin, planar dielectric nanostructures. Most notably, we experimentally achieve near-unity circular dichroism with ~90% of the light with the chosen helicity being transmitted at a wavelength of 540 nm. This is the highest value demonstrated to date for any geometry in the visible spectrum. We interpret this result within the charge-current multipole expansion framework and show that the excitation of higher-order multipoles is responsible for the giant circular dichroism. These experimental results enable the realization of high-performance miniaturized chiro-optical components in a scalable manner at optical frequencies.

## Introduction

An object is said to be chiral if it cannot be superposed with its mirror image via rotation or translation operations alone. This geometric or structural chirality is an intrinsic part of the natural world and manifests itself in numerous forms, ranging from molecules like amino acids^1^ to macroscale objects such as quartz crystals^2^ and even entire living organisms^3^. Due to its profound implications for various disciplines, particularly pharmaceutics^4^, the study and manipulation of chiral media has been a highly active field of study in recent years.

In the context of optics, chiral objects interact with circularly polarized (CP) light in different ways depending on their handedness; they are said to exhibit optical chirality and are characterized by circular dichroism and circular birefringence. The latter is also known as optical activity. However, chiro-optical responses in most naturally occurring compounds are very small due to the fundamental mismatch in size scale between molecules and the wavelength of incident light.

Suitably designed three-dimensional (3D) metamaterials^5, 6, 7, 8^ comprising arrays of chiral nanostructures possess chiro-optical responses several orders of magnitude greater than their naturally occurring counterparts^9, 10, 11, 12, 13, 14, 15, 16, 17, 18, 19, 20^. Circular dichroism on the order of several tens of percent, as well as negative refractive indices^13, 14, 15, 16^ due to chirality have been observed at THz and GHz frequencies. However, complexities associated with 3D fabrication and a general lack of suitable, low-loss materials at optical wavelengths have so far limited their application.

The planar counterparts^21, 22^ to these chiral metamaterials, chiral metasurfaces^23, 24, 25, 26, 27, 28, 29, 30, 31, 32, 33^, overcome these challenges but exhibit chiro-optical responses that are significantly weaker and become vanishingly small close to normal incidence. This is due to fundamental symmetry considerations: in planar structures, there always exists a plane of reflection symmetry perpendicular to the surface normal which renders them geometrically achiral. Symmetry-breaking effects due to the substrate are known to be negligible in terms of their contribution to the strength of the eventual chiral effect^34^. As such, one approach to achieve strong optical chirality is to vary the material composition such as in quasi-3D multilayered or oligomer structures^23, 26^. An alternative method that stays true to the planar geometry is to break the symmetry via external experimental configurations, that is, illuminating at oblique incidence and observing the resultant zeroth-order beam^28, 29^. One can also observe a chiral response in higher orders of diffracted light from planar chiral structures at normal incidence^30^. Although these planar structures can lead to exotic chiro-optical effects such as circular conversion dichroism and asymmetric transmission^31, 32^ due to the reversal of their handedness when illuminated from opposite sides, it is crucial to note that they remain fundamentally distinct from the ‘true’ geometric chirality characteristic of 3D objects; following established nomenclature^24^, we call the former ‘extrinsic chirality’ and its 3D counterpart ‘intrinsic chirality’.

**Figure 1:**
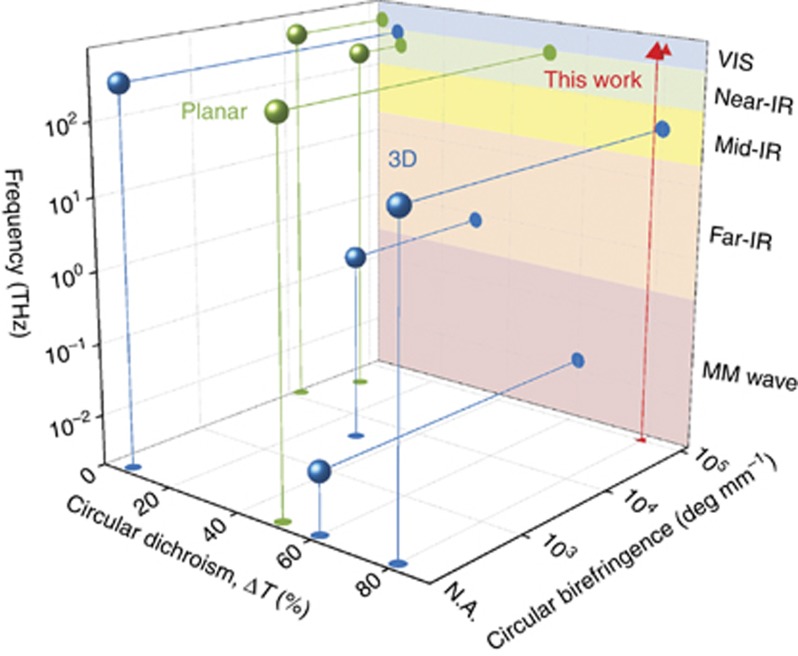
Typical chiral metamaterials. Representative examples of chiral metamaterials of varying designs and their measured performances at their respective operating frequencies. The performances are characterized by their circular dichroism (unnormalized, calculated from the differential transmittance Δ*T*) and circular birefringence, where applicable. Different colors correspond to different geometries of the chiral structures, that is, 3D (blue)^9, 11, 12, 26^ and planar (green)^25, 29, 33^. Generally, the former possess significantly stronger chiro-optical effects due to intrinsic chirality. The experimental results from this work using planar structures that exhibit intrinsic chirality are represented by the red triangle.

In this work, we overcome these inherent limitations associated with chiral metasurfaces by using planar dielectric gammadion nanostructures whose radiation patterns are dominated by exceptionally strong electric and magnetic higher-order multipole responses in the visible spectrum. Contrary to conventional wisdom, the chiro-optical response of these planar structures is characterized by *intrinsic* chirality; it manifests strongly at normal incidence and does not change when the incidence direction is reversed. Numerical simulations show that one can obtain close to unity circular dichroism in transmission, with more than 95% of incident light of a chosen helicity being transmitted at ~540 nm; experimentally we achieve ~80% circular dichroism and circular birefringence exceeding 100 000°/mm (in units of polarization rotation per unit thickness). These values are comparable to or exceeding 3D geometrically chiral metamaterials (Figure 1), which is unprecedented for planar structures. In this comparison, we have used the un-normalized circular dichroism values given by the raw differential transmittance (i.e., transmitted power) to ensure an accurate representation of device performance.

To date, gammadion structures have been shown to possess circular dichroism on the order of a few percent^26, 27, 33^. Other works involving planar structures have made use of the interaction between electric dipole and quadrupolar modes to realize chiro-optical behavior and high-quality factor modes in the mid-infrared regime^35^, but its anisotropic nature results in non-negligible circular polarization conversion such that the measured output cannot be attributed to a purely chiral response. In our design, the motif and all its relevant geometrical parameters are optimized to ensure the absence of circular polarization conversion and that higher-order multipoles (up to the magnetic octupole) are dominant. This engineering of the multipoles results in unprecedented performance values in terms of near-unity transmission and circular dichroism, particularly in the visible spectrum, and thus showcases the full potential of planar chiral nanostructures.

## Materials and methods

**Figure 2:**
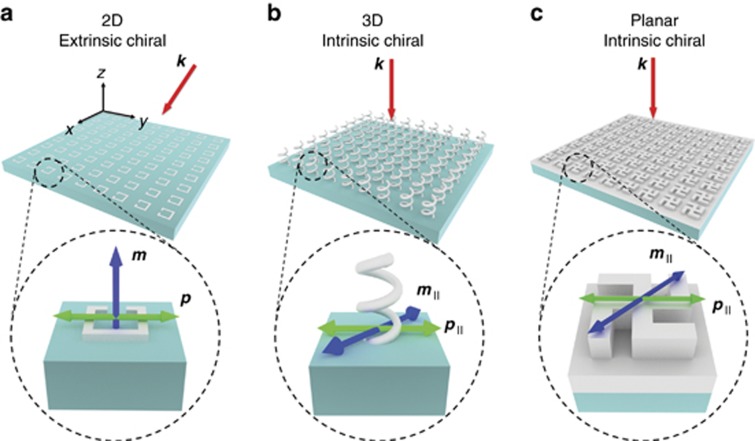
Origins of optical chirality. (**a**–**c**) Schematic illustrations of the operating principle of a representative **a** planar extrinsic (pseudo) chiral metasurface, **b** 3D intrinsic metamaterial and **c** optically thick planar structure with intrinsic chirality under their respective illumination conditions (red arrows). The insets show magnified views of the structures overlaid with the relative orientations of electric (***p***, green arrow) and magnetic (***m***, blue arrow) multipole moments generated under such illumination. These moments are drawn as simple vector arrows for ease of illustration. In **a**, due to geometrical constraints, the net electric and magnetic moments are always orthogonal. In **b** and **c**, the chiral structures can support in-plane magnetic moments (***m***_∥_) that have a non-zero component along the direction of the in-plane electric moment (***p***_∥_).

We first look at the origin of chiro-optical responses in various metamaterials in greater detail. From an electromagnetic source perspective, this can be understood by considering the superposition of radiated electric fields from electric and magnetic multipole moments generated in a structure upon illumination (Figure 2a–2c). When these moments are perpendicular to each other and lie on two orthogonal planes, optical activity in the form of polarization rotation can only occur at oblique angles, where the radiated fields are non-parallel. On the other hand, coplanar, orthogonal moments have parallel electric field components and thus no rotation of the field can occur. As a result, at least some components of electric and magnetic moments that are parallel to each other must exist, such that their resultant electric fields are aligned perpendicularly, leading to polarization rotation and hence optical activity. This intuitive picture is a generalization (to oblique incidence) of what is known as the Rosenfeld criterion^36^ in the relevant literature, which states that the condition for chiro-optical activity can be written as ***p*·*m***≠0, where ***p*** and ***m*** are the net electric and magnetic dipole moments, respectively. Conventional methods of achieving strong chiral responses thus involve the use of either planar nanostructures under oblique incidence or intrinsically chiral metamaterials (Figure 2a and 2b). In the former, due to the planar geometry and deeply subwavelength thickness only tangential currents exist, and the resultant net magnetic moment (***m***) therefore always points out of plane, orthogonal to the in-plane net electric moment (***p***) (Figure 2a). Consequently, the required superposition of radiated fields can only occur off-normal (where the radiation from ***m*** is non-zero). This is true regardless of whether chiral or achiral shapes are used. While in principle the presence of a substrate should break the symmetry, such effects are known to be negligible in terms of magnitude^34^. In contrast, for 3D chiral structures, the geometry admits both normal and tangential currents, leading to the generation of in-plane magnetic moments (***m***_∥_) with a non-zero component parallel to the in-plane electric moment (***p***_∥_), resulting in strong chiro-optical behavior at normal incidence (Figure 2b).

A natural extension to this concept would be to consider the possibility of in-plane magnetic moments existing in *planar* chiral structures. For this to occur, the structures need to possess a finite thickness on the order of the excitation wavelength in the material and, in the case of dielectric materials, have sufficiently high refractive indices to support out-of-plane electric displacement currents that generate in-plane magnetic moments^37^ (Figure 2c). Further details on the physical origins of such modes are provided in Supplementary Fig. S1 and the accompanying text. In this case, the resultant chiro-optical response would be characteristic of intrinsic chirality, that is, it can be observed at normal incidence and does not change when the direction of incidence is reversed. This is a restatement of the fact that extrinsically chiral objects (Figure 2a) change their handedness or sense of twist depending on the observer’s perspective, whereas intrinsically chiral ones do not (Figure 2b). Although the existence of these in-plane magnetic moments in high-index dielectrics such as silicon has been well studied using achiral nanoparticles of various geometries to realize highly directional nanoantennas and Huygen’s metasurfaces^38, 39, 40, 41, 42, 43^, their chiral counterparts have only been superficially explored. We emphasize that these planar structures are fundamentally distinct from 3D intrinsically chiral objects (such as the helix) as they still possess a plane of reflection symmetry in the normal direction, and a traditional symmetry argument^44^ would therefore suggest that they cannot exhibit intrinsic chiral behavior regardless of thickness and refractive index. This neglects the possibility for vertical, out-of-plane electric displacement currents in planar geometries due to propagation effects.

**Figure 3:**
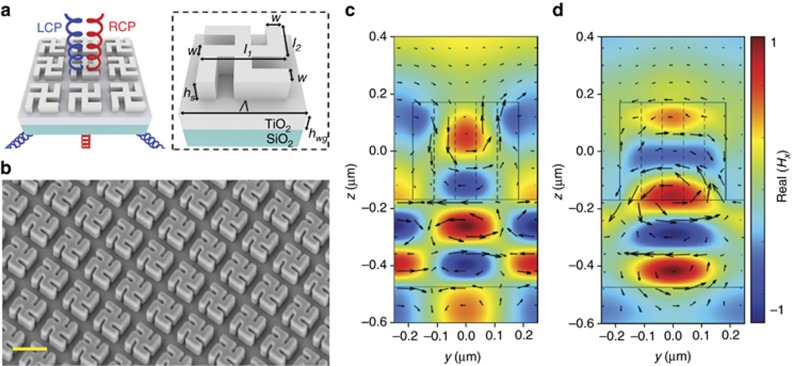
Design and simulation of gammadion nanostructures. (**a**, Left) Schematic of the optical response of the planar intrinsic chiral device comprising of TiO_2_ gammadions overlaid on a TiO_2_ thin film on a glass substrate. It transmits normally incident RCP light (red helix) in the zeroth order while diffracting LCP light (blue helix) into the first order. (**a**, Right) The design parameters are *w*=74 nm, *l*_1_=370 nm, *l*_2_=220 nm, *h*_s_=340 nm and *h*_wg_=300 nm. The unit cell size *Λ* is 500 nm. (**b**) Tilted-view scanning electron micrograph of the fabricated structures. Scale bar: 500 nm. (**c**, **d**) *y*−*z* cut-plane showing the simulated in-plane magnetic field ***H***_*x*_ (color plot) and the associated current distribution ***J***_*z*_*+**J***_*y*_ (black arrows) in the gammadion structures under **c** LCP and **d** RCP incidence. The rectangular outline from *z*=0.17 μm to *z*=−0.17 μm represents the gammadion structure, with subsequent layers below being the TiO_2_ waveguide and substrate respectively. The difference in the number of antinodes of ***H***_*x*_ within the structure illustrates its chiral behavior due to different multipoles when illuminated with different circular polarization states.

We use a common motif, the gammadion, as our planar chiral nanostructures (Figure 3a and 3b). Periodic arrays are fabricated using electron beam lithography and atomic layer deposition of titanium oxide, a relatively high-index dielectric lossless in the visible spectrum^45^. Intuitively, their chiral behavior arises from the orthogonal arrangement of arms with respect to each other and the resulting linear polarization conversion (Figures 2c and 3a); excitation with, for example, *x*-polarized light results in an induced displacement current and radiated electric fields along the *y* axis due to the geometry of the structure. Note that the choice of these structures with fourfold (*C*_4_) symmetry ensures the absence of linear birefringence, that is, the amplitude transmission coefficients are related by *t*_*xx*_=*t*_*yy*_, *t*_*yx*_=−*t*_*xy*_, such that circular polarization conversion into both right circularly polarized (RCP) and left circularly polarized (LCP) (*t*_*xx*_−*t*_*yy*_±*i*(*t*_*xy*_+*t*_*yx*_), respectively) is zero. The latter quantity is distinct from but can be often misconstrued as circular dichroism. Additionally, we note that at normal incidence for *C*_4_ symmetric structures, reciprocity ensures that there is no dichroism in reflection^27^, and as a result, diffraction (into higher orders) must exist in order for dichroism to be observed in transmission. In the design of our nanostructure array, the periodicity *Λ* (Figure 3a and 3b) was chosen to be 500 nm, such that the targeted free space wavelength (540 nm) is between the periodicity and effective wavelength in the substrate.

The performance of our structure under RCP and LCP light at normal incidence is schematically illustrated in Figure 3a; RCP light transmits in the zeroth order, whereas LCP light is completely diffracted into the first order (here 46°). The angle at which this occurs can be controlled by adjusting the periodicity. Here, we have included a waveguide layer of thickness 300 nm to further increase the circular dichroism in the zeroth-order transmission, analogous to the design of guided or leaky mode resonance structures^46^. Essentially, at resonance, part of the incident wave of the chosen helicity (RCP) is coupled into the guided mode, which then slowly leaks from the waveguide and interferes with the applied wave to produce a sharp filtering response. For the other helicity (LCP) due to chirality of the gammadion structure, the effective index of the mode is different, and thus, light does not couple efficiently into the waveguide. As a result, the structure exhibits a background response typical of the multilayered geometry. The same is true for other wavelengths away from the resonance. Further details, including the optimization of performance with and without the waveguide layer, can be found in Supplementary Fig. S2−S4. However, we emphasize that the structure is not reliant upon this waveguide layer in order to exhibit its chiro-optical behavior; the origin of this response can be traced back to the electromagnetic modes (multipoles) excited purely within the structure. It is readily apparent from Supplementary Fig. S4 that field profiles similar to Figure 3 exist within the structure, even without the waveguide layer. The latter exists only to improve overall transmission efficiency and circular dichroism by modulating the transmission envelope.

A closer analysis of these modes excited in the gammadion reveals the presence of higher-order multipole moments. Using finite-difference time-domain simulations (Lumerical Inc.), we computed the in-plane component of the magnetic fields (here ***H***_*x*_) and the vector sum of corresponding generating currents (***J***_*z*_ and ***J***_*y*_) for both incident circular polarization states across a vertical cut through the middle of the structure (*y*–*z* plane) at 540 nm (Figure 3c and 3d). For the RCP case, ***H***_*x*_ exhibits three antinodes opposite in sign within the structure, indicative of an octupole dominated response (Figure 3d). In contrast, under LCP illumination at the same wavelength, fewer antinodes of opposite sign are observed (Figure 3c), characteristic of a mixture of lower-order multipole (quadrupolar) moments. This is a direct result of the chirality of the structure, which leads to significantly different current distributions and far-field radiation patterns for the two circular polarization states. As mentioned in the previous section, these field distributions remain the same even without the underlying TiO_2_ waveguide layer, indicating that the chirality arises from the planar structures themselves. It is important to note that the use of higher-order multipoles is essential to achieve a strong chiro-optical response due to the diffraction requirement for chirality in this configuration. Although engineering only the dipolar response is sufficient for achiral, subwavelength devices such as Huygen’s metasurfaces operating in the zeroth order, manipulation of the radiation into large angles is necessary here. One should therefore make use of several higher-order multipoles, whose primary radiation directions are off-normal.

To study the contributions of each multipole in more detail, we performed multipolar decomposition using the charge-current expansion framework to represent the origin of electromagnetic radiation in the structure by point-like multipole sources^47, 48^. We considered three families of multipole excitation: electric, magnetic, and toroidal and performed the expansion up to the electric/magnetic octupole and toroidal quadrupole. Toroidal excitations can be treated explicitly within such a charge-current expansion framework, distinct from the usual far-field expansion using vector spherical harmonics that are comprised only of electric and magnetic coefficients. They comprise a family of elementary electromagnetic sources distinct from their electric and magnetic counterparts, that is, they are associated with radial current density of the form **r·J**, in contrast to charge and azimuthal current density (**r × J**), which correspond to electric and magnetic multipole moments, respectively^47, 48, 49^. These toroidal moments have drawn significant attention recently by being responsible for exotic phenomena such as non-radiating anapoles^50^ and have spurred research into novel devices ranging from toroidal lasers to sensors^49^.

**Figure 4:**
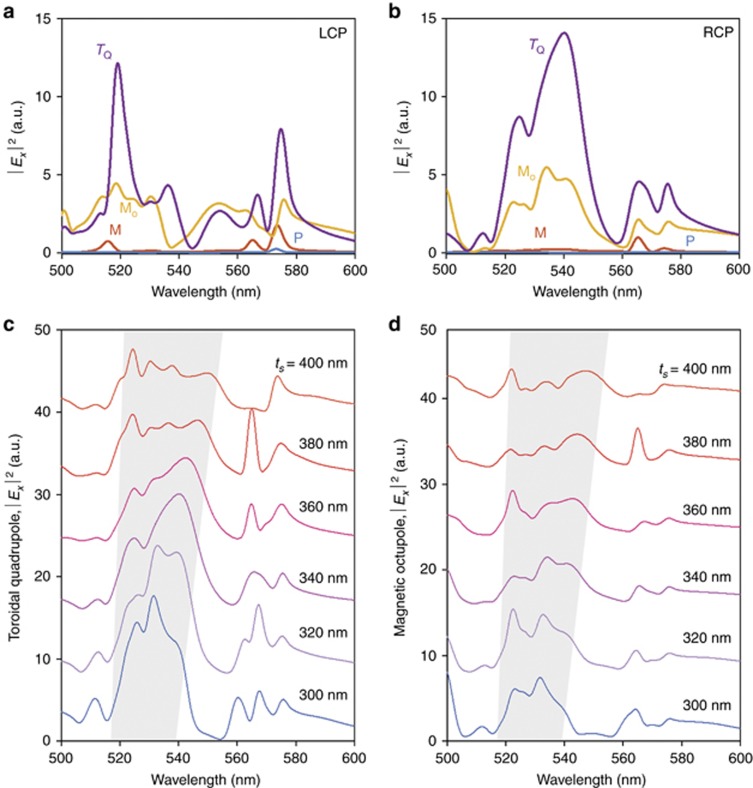
Multipole decomposition. (**a**, **b**) The far-field intensity |*E*_*x*_|^2^ with explicit contributions from each multipole for **a** LCP and **b** RCP incidence. Only the two strongest contributions, the toroidal quadrupole (*T*_Q_, purple) and magnetic octupole (*M*_O_, yellow), as well as the standard electric (*P*, cyan) and magnetic dipole responses (*M*, red) are shown for clarity. (**c**, **d**) Tuning of the spectral position and strength of the **c** toroidal quadrupole and **d** magnetic octupole response by changing the aspect ratio of the structure. Here, the length and width of each arm of the gammadion were kept constant at 370 and 74 nm, respectively, while the height was varied from 300 to 400 nm. Each curve is offset by 8 units along the *y* axis. The shaded regions indicate the region of interest, where the resonances are tuned with height of gammadion.

Intensities of the zeroth-order radiated electric field |*E*_*x*_|^2^ for a single gammadion structure (in the presence of its neighbors) for various multipoles are shown in Figure 4a and 4b. Here, the excitation light source was CP and normally incident. Only the two strongest contributions as well as traditional electric and magnetic dipolar responses are shown for clarity. A complete set of multipole responses is provided in Supplementary Fig. S5. We observe strong dichroism in the zeroth-order electric field at ~540 nm, where the higher-order multipole radiation peaks for RCP but exhibits a strong dip for LCP. As expected from the field distribution shown in Figure 3d, the magnetic octupole plays a large role in the chiro-optical response under RCP illumination. However, it is revealed that the dominant factor is actually the toroidal quadrupole (Figure 4b). This affirms the importance of considering toroidal moments as an independent source of radiation (rather than subsumed under the electric multipole)^12^. It is also interesting to note that the electric and magnetic dipole contributions in this case are very much weaker than their higher-order counterparts; they are almost negligible for both input polarizations, which affirms chirality as a concept beyond the usual dipolar approximation^30, 36^. This provides further evidence of the importance of generating and tailoring higher-order multipole responses in the design of metamaterials and metasurfaces in general.

We emphasize that these strong high-order multipolar contributions are only possible due to the geometry of the structure; the thickness (on the order of the incident wavelength) supports in-plane magnetic moments necessary for generating magnetic and toroidal responses, whereas the proximity of the individual ‘arms’ in the gammadion enables interactions between the induced charges and currents, thereby giving rise to higher-order multipoles. This provides an intuitive picture for how the overall strength of the multipoles present in the system can be tuned by varying both the length of the individual arms and the thickness, that is, by changing the aspect ratio. This is analogous to the tuning of magnetic and electric dipoles shown earlier in Supplementary Fig. S1. Here, in Figure 4c and 4d, by varying the height for a fixed length and width of the gammadion (370 and 74 nm, respectively), we show that a similar relationship between aspect ratio and multipole strength holds true. Note that in order to achieve large chiro-optical responses, the phases of the multipoles must also be considered such that their radiation interferes constructively (destructively) under RCP (LCP) illumination; hence, the optimal thickness was chosen to be 340 nm instead of the lower values that appear to have larger multipole strength but smaller overall zeroth-order transmittance. This fact is verified in Supplementary Fig. S6, where the overlap between the dominant multipole radiation and simulated net zeroth-order transmittance as well as a color plot of the circular dichroism are shown.

**Figure 5:**
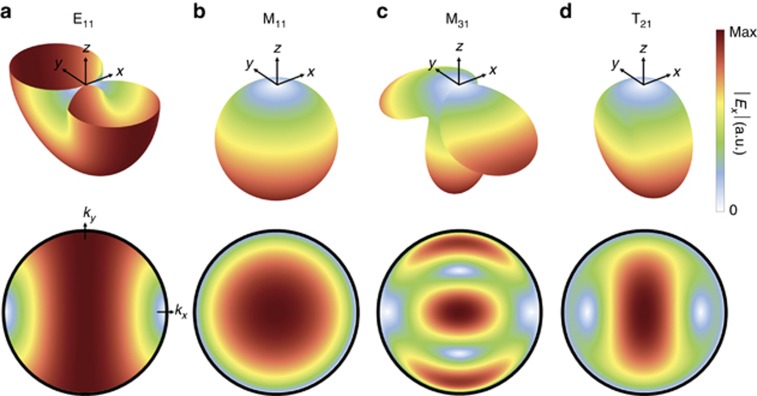
Far-field angular radiation patterns (|*E*_*x*_|^2^). (**a**–**d**, Top) 3D far-field angular radiation patterns and (**a**–**d**, bottom) their 2D projections for the **a** electric dipole (*E*_11_), (**b**) magnetic dipole (*M*_11_), (**c**) magnetic octupole (*M*_31_) and (**d**) toroidal quadrupole (*T*_21_), respectively. For clarity, only the forward radiation direction (*z*≤0 half-space) is shown. The subscripts denote the corresponding spherical harmonic, *Y_lm_*, used to calculate the electric field. Here, the *m*=+1 harmonic is chosen as it is the dominant contribution to radiation along the *z*-direction, corresponding to zeroth-order transmission from the structure. Each point on the 2D projections obeys *k*_*x*_^2^+*k*_*y*_^2^+*k*_*z*_^2^=*k*_0_^2^ (*k*_0_=2π/λ is the wavevector), which defines the radiation direction, and the magnitude is given by the color bar. The black lines demarcate where *k*_*z*_=0, that is, the radiation only occurs in the *x–y* plane.

Figure 5 illustrates the 3D and projected 2D angular radiation patterns (|*E*_*x*_|) of both conventional dipoles and dominant multipoles that contribute to zeroth-order radiation in the structure. Here, they are expressed in the spherical harmonic basis (instead of Cartesian), following the approach used in Ref. 47 and are classified by their source (electric (*E*), magnetic (*M*) and toroidal (*T*)) as well as a pair of indices *lm* specifying the order and degree of the spherical harmonic being considered. Note that dipole, quadrupole and octupole modes correspond to *l*=1, 2 and 3, respectively, and their associated *m* values range from *−l* to *l*. Since only *m*=±1 harmonics have non-vanishing contributions to the zeroth order and the radiation patterns from *m*=+1 and −1 are identical, in Figure 5 we show only the case for *m*=+1. Additionally, in the 3D figures, only the forward scattered (transmitted) radiation corresponding to the *z*≤0 half-space is illustrated for clarity. The 2D projections are analogous to what would be seen upon capturing the 3D radiation patterns with a microscope objective and imaging its back focal plane. The axes correspond to the normalized wavenumbers (*k*_*x*_/*k*_0_ and *k*_*y*_/*k*_0_) such that every point on the image satisfies *k*_*x*_^2^+*k*_*y*_^2^+*k*_*z*_^2^=*k*_0_^2^. This defines the direction of radiation, and the colormap specifies its magnitude. From these plots, one sees that it is the difference in intensities of the *M*_31_ and *T*_21_ modes that lead to the large circular dichroism in the zeroth-order radiation under RCP (LCP) illumination of the structure.

## Results and discussion

**Figure 6:**
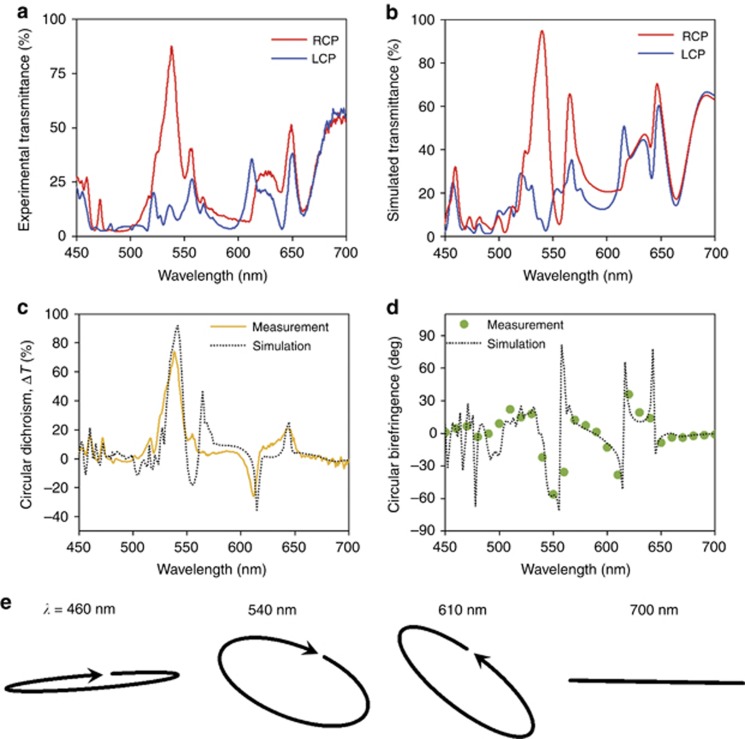
Characterization of gammadion performance. (**a**, **b**) Experimental **a** and simulated **b** zeroth-order transmittance spectra for the planar chiral nanostructures for both RCP (red) and LCP incident light (blue). (**c**) Experimental (solid line) and simulated (dotted line) circular dichroism spectra, defined as the differential transmittance between RCP and LCP from the results shown in **a** and **b**, respectively. (**d**) Circular birefringence spectra (also known as the optical rotary dispersion) of the gammadions, comprising azimuth rotation angles from polarimetric data (green circles) and finite-difference time-domain (FDTD) calculations (dotted line). (**e**) Polarization ellipses of the zeroth-order transmitted polarization states at various wavelengths under linearly polarized incidence. Their ellipticity and azimuthal angles are obtained from the experimental data shown in **c**, **d**.

We experimentally characterized the chiro-optical properties of our planar nanostructures using a home-built microscopy setup. The light source is a supercontinuum laser, and the detectors used in turn are a commercial spectrometer and polarimeter. Figure 6a and 6b illustrates the measured and simulated zeroth-order transmittance for both RCP and LCP light across the visible spectrum (450–700 nm). Note that these values are normalized to the transmitted light through an unpatterned glass substrate (with the TiO_2_ layer) to remove contributions from reflection due to the substrate as well as interference effects caused by the TiO_2_ thin film. The unnormalized circular dichroism spectrum given by the raw differential transmittance (Δ*T*) is shown in Figure 6c. We observe that ~87% of RCP light is transmitted in the zeroth order, with a difference in transmittance (i.e., circular dichroism) of almost 80% at a wavelength of 540 nm. Incident LCP light is almost completely diffracted into the first order (here 46°) by design as dichroism in reflection is forbidden by the *C*_4_ symmetry and there is no absorption. These values agree well with the simulations; a slight discrepancy of <10% is likely due to structural imperfections during fabrication, which mainly arise from proximity effects during electron beam lithography and deviations in the resist thickness. These could lead to resultant band broadening and lower efficiencies. It should also be noted that even in the simulations, residual reflection (~5%) remains after accounting for substrate effects: this occurs since the structures used in the current scheme lack the sufficient degrees of freedom to be impedance matched to free space after optimizing for chiro-optical behavior. In principle, this can be overcome with the use of more complicated structures using multiple coupled resonances. In addition, some of the spectral features are below the resolution of the spectrometer (~1.5 to 2 nm), such as those at 560 and 610 nm in Figure 6a and 6b, resulting in them being truncated and thus being different from the simulated results. As is characteristic of an intrinsically chiral response, besides exhibiting strong chiral behavior at normal incidence, the handedness of the structure is also invariant to the propagation direction of light (see Supplementary Fig. S7)—when the sample is reversed, the results are identical (to within experimental noise), as shown in Figure 6a and 6b.

The circular birefringence was measured by performing polarimetric measurements with linearly polarized input; the azimuthal angle of the (generally elliptical) output polarization state corresponds to the polarization rotation due to circular birefringence. Figure 6d shows the resultant rotation as a function of wavelength (also known as the optical rotary dispersion). Note that due to the minimum bandwidth achievable for the supercontinuum laser (~5 nm) and the lack of spectroscopic capabilities in the polarimeter, sharp features in the spectrum cannot be resolved. However, by overlaying simulated results with experimental data, we observe close agreement overall. Furthermore, a peak polarization rotation of ~60° for a combined structure and waveguide thickness of ~600 nm is achieved (~100 000°/mm, in units of degrees of rotation per unit length). To put this result in perspective, the polarization rotation of most naturally occurring chiral media is on the order of degrees per mm; to the best of our knowledge, this is also significantly larger than state-of-the-art chiral metasurfaces, including those operating at oblique incidence (Figure 1).

## Conclusions

Recent advances in the field of metamaterials have led to further reductions in device size, complexity and fabrication costs in the form of metasurfaces. This has in turn enabled a wide variety of planar optical devices such as lenses^51, 52^, holograms^53^ and q-plates^54^ as well as digital or programmable metmaterials^55, 56, 57^ that center on the principles of phase and amplitude modulation. For true polarization control at the level of a single nanostructure, chiral structures are indispensable. Although there has been much seminal work done in this area by the community, much of it has centered on either extrinsic or lossy plasmonic devices or demonstrated only at GHz/THz frequencies, where the size scales make it possible to engineer various 3D, complex structures. Crucially, there is a lack of compelling demonstrations of high-performance planar chiral devices at optical wavelengths. Moreover, certain subtleties regarding the underlying distinction between planar and 3D intrinsic chirality have yet to be made explicit. Here, we have demonstrated planar chiral nanostructures with intrinsic chirality by using the propagation effect and measure close to unity circular dichroism (80%) and giant polarization rotation (100 000°/mm) at normal incidence for the visible spectrum. This was achieved by tailoring the higher-order multipole response, which plays a significant role in off-normal scattering compared to the usual dipolar approximation used to characterize chiral phenomena. We expect that this could pave the way for the widespread adoption of planar chiral nanostructures in numerous applications ranging from polarization optics to telecommunications, given their single step lithographic nature and easily scalable fabrication process.

## References

[bib1] Le Bel JA. Sur les relations qui existent entre les formules atomiques des corps organiques et le pouvoir rotatoire de leurs dissolutions. Bull Soc Chim 1874; 22: 337–347.

[bib2] Hazen RM, Sholl DS. Chiral selection on inorganic crystalline surfaces. Nat Mater 2003; 2: 367–374. 12776102 10.1038/nmat879

[bib3] Sharma V, Crne M, Park JO, Srinivasarao M. Structural origin of circularly polarized iridescence in jeweled beetles. Science 2009; 325: 449–451. 19628862 10.1126/science.1172051

[bib4] Pályi G, Zucchi C, Caglioti L. Advances in Biochirality. Amsterdam: Elsevier; 1999.

[bib5] Boltasseva A, Atwater HA. Low-loss plasmonic metamaterials. Science 2011; 331: 290–291. 21252335 10.1126/science.1198258

[bib6] Shalaev VM. Optical negative-index metamaterials. Nat Photonics 2007; 1: 41–48.

[bib7] Soukoulis CM, Wegener M. Optical metamaterials—more bulky and less lossy. Science 2010; 330: 1633–1634. 21164003 10.1126/science.1198858

[bib8] Soukoulis CM, Wegener M. Past achievements and future challenges in the development of three-dimensional photonic metamaterials. Nat Photonics 2011; 5: 523–530.

[bib9] Zhou JF, Chowdhury DR, Zhao RK, Azad AK, Chen H-T et al. Terahertz chiral metamaterials with giant and dynamically tunable optical activity. Phys Rev B 2012; 86: 035448.

[bib10] Decker M, Zhao R, Soukoulis CM, Linden S, Wegener M. Twisted split-ring-resonator photonic metamaterial with huge optical activity. Opt Lett 2010; 35: 1593–1595. 20479819 10.1364/OL.35.001593

[bib11] Gansel JK, Thiel M, Rill MS, Decker M, Bade K et al. Gold helix photonic metamaterial as broadband circular polarizer. Science 2009; 325: 1513–1515. 19696310 10.1126/science.1177031

[bib12] Raybould T, Fedotov VA, Papasimakis N, Kuprov I, Youngs IJ et al. Toroidal circular dichroism. Phys Rev B 2016; 94: 035119.

[bib13] Plum E, Zhou J, Dong J, Fedotov VA, Koschny T et al. Metamaterial with negative index due to chirality. Phys Rev B 2009; 79: 035407.

[bib14] Zhou JF, Dong JF, Wang BN, Koschny T, Kafesaki M et al. Negative refractive index due to chirality. Phys Rev B 2009; 79: 121104.

[bib15] Zhang S, Park Y-S, Li J, Lu XC, Zhang WL et al. Negative refractive index in chiral metamaterials. Phys Rev Lett 2009; 102: 023901. 19257274 10.1103/PhysRevLett.102.023901

[bib16] Wang BN, Zhou JF, Koschny T, Soukoulis CM. Nonplanar chiral metamaterials with negative index. Appl Phys Lett 2009; 94: 151112.

[bib17] Liu N, Liu H, Zhu SN, Giessen H. Stereometamaterials. Nat Photonics 2009; 3: 157–162.

[bib18] Kuzyk A, Schreiber R, Fan ZY, Pardatscher G, Roller E-M et al. DNA-based self-assembly of chiral plasmonic nanostructures with tailored optical response. Nature 2012; 483: 311–314. 22422265 10.1038/nature10889

[bib19] Govorov AO, Fan ZY, Hernandez P, Slocik JM, Naik RR. Theory of circular dichroism of nanomaterials comprising chiral molecules and nanocrystals: plasmon enhancement, dipole interactions, and dielectric effects. Nano Lett 2010; 10: 1374–1382. 20184381 10.1021/nl100010v

[bib20] Engheta N. Special issue on wave interactions with chiral and complex media. J Electromagn Waves Appl 1992; 6: 537–793.

[bib21] Kildishev AV, Boltasseva A, Shalaev VM. Planar photonics with metasurfaces. Science 2013; 339: 1232009. 23493714 10.1126/science.1232009

[bib22] Yu NF, Capasso F. Flat optics with designer metasurfaces. Nat Mater 2014; 13: 139–150. 24452357 10.1038/nmat3839

[bib23] Banzer P, Woźniak P, Mick U, De Leon I, Boyd RW. Chiral optical response of planar and symmetric nanotrimers enabled by heteromaterial selection. Nat Commun 2016; 7: 13117. 27734960 10.1038/ncomms13117 PMC5065623

[bib24] Plum E, Fedotov VA, Zheludev NI. Optical activity in extrinsically chiral metamaterial. Appl Phys Lett 2008; 93: 191911.

[bib25] Plum E, Liu XX, Fedotov VA, Chen Y, Tsai DP et al. Metamaterials: optical activity without chirality. Phys Rev Lett 2009; 102: 113902. 19392202 10.1103/PhysRevLett.102.113902

[bib26] Decker M, Klein MW, Wegener M, Linden S. Circular dichroism of planar chiral magnetic metamaterials. Opt Lett 2007; 32: 856–858. 17339960 10.1364/ol.32.000856

[bib27] Bai BF, Svirko Y, Turunen J, Vallius T. Optical activity in planar chiral metamaterials: theoretical study. Phys Rev A 2007; 76: 023811.

[bib28] De Leon I, Horton MJ, Schulz SA, Upham J, Banzer P et al. Strong, spectrally-tunable chirality in diffractive metasurfaces. Sci Rep 2015; 5: 13034. 26338445 10.1038/srep13034 PMC4559672

[bib29] Papakostas A, Potts A, Bagnall DM, Prosvirnin SL, Coles HJ et al. Optical manifestations of planar chirality. Phys Rev Lett 2003; 90: 107404. 12689032 10.1103/PhysRevLett.90.107404

[bib30] Klimov V, Zabkov IV, Pavlov AA, Shiu R-C, Chan H-C et al. Manipulation of polarization and spatial properties of light beams with chiral metafilms. Opt Express 2016; 24: 6172–6185. 27136811 10.1364/OE.24.006172

[bib31] Fedotov VA, Mladyonov PL, Prosvirnin SL, Rogacheva AV, Chen Y et al. Asymmetric propagation of electromagnetic waves through a planar chiral structure. Phys Rev Lett 2006; 97: 167401. 17155432 10.1103/PhysRevLett.97.167401

[bib32] Singh R, Plum E, Menzel C, Rockstuhl C, Azad AK et al. Terahertz metamaterial with asymmetric transmission. Phys Rev B 2009; 80: 153104.

[bib33] Kuwata-Gonokami M, Saito N, Ino Y, Kauranen M, Jefimovs K et al. Giant optical activity in quasi-two-dimensional planar nanostructures. Phys Rev Lett 2005; 95: 227401. 16384264 10.1103/PhysRevLett.95.227401

[bib34] Zhao RK, Koschny T, Soukoulis CM. Chiral metamaterials: retrieval of the effective parameters with and without substrate. Opt Express 2010; 18: 14553–14567. 20639941 10.1364/OE.18.014553

[bib35] Wu CH, Arju N, Kelp G, Fan JA, Dominguez J et al. Spectrally selective chiral silicon metasurfaces based on infrared fano resonances. Nat Commun 2014; 5: 3892. 24861488 10.1038/ncomms4892

[bib36] Schäferling M. Chiral Nanophotonics. Switzerland: Springer International Publishing; 2017.

[bib37] Kuznetsov AI, Miroshnichenko AE, Fu YH, Zhang JB, Luk’yanchuk B. Magnetic light. Sci Rep 2012; 2: 492. 22768382 10.1038/srep00492 PMC3389365

[bib38] Staude I, Miroshnichenko AE, Decker M, Fofang NT, Liu S et al. Tailoring directional scattering through magnetic and electric resonances in subwavelength silicon nanodisks. ACS Nano 2013; 7: 7824–7832. 23952969 10.1021/nn402736f

[bib39] Decker M, Staude I, Falkner M, Dominguez J, Neshev DN et al. High-efficiency dielectric Huygens’ surfaces. Adv Opt Mater 2015; 3: 813–820.

[bib40] Pfeiffer C, Grbic A. Metamaterial Huygens’ surfaces: tailoring wave fronts with reflectionless sheets. Phys Rev Lett 2013; 110: 197401. 23705738 10.1103/PhysRevLett.110.197401

[bib41] Fu YH, Kuznetsov AI, Miroshnichenko AE, Yu YF, Luk’yanchuk B. Directional visible light scattering by silicon nanoparticles. Nat Commun 2013; 4: 1527. 23443555 10.1038/ncomms2538

[bib42] Miroshnichenko AE, Kivshar YS. Fano resonances in all-dielectric oligomers. Nano Lett 2012; 12: 6459–6463. 23170879 10.1021/nl303927q

[bib43] Krasnok AE, Miroshnichenko AE, Belov PA, Kivshar YS. All-dielectric optical nanoantennas. Opt Express 2012; 20: 20599–20604. 23037107 10.1364/OE.20.020599

[bib44] Menzel C, Rockstuhl C, Lederer F. Advanced jones calculus for the classification of periodic metamaterials. Phys Rev A 2010; 82: 053811.

[bib45] Devlin RC, Khorasaninejad M, Chen WT, Oh J, Capasso F. Broadband high-efficiency dielectric metasurfaces for the visible spectrum. Proc Natl Acad Sci USA 2016; 113: 11473–11478. 10.1073/pnas.1611740113 PMC5035844 27601634

[bib46] Wang SS, Magnusson R. Theory and applications of guided-mode resonance filters. Appl Opt 1993; 32: 2606–2613. 20820422 10.1364/AO.32.002606

[bib47] Savinov V, Fedotov VA, Zheludev NI. Toroidal dipolar excitation and macroscopic electromagnetic properties of metamaterials. Phys Rev B 2014; 89: 205112.

[bib48] Radescu EE, Vaman G. Toroid moments in the momentum and angular momentum loss by a radiating arbitrary source. Phys Rev E 2002; 65: 035601. 10.1103/PhysRevE.65.035601 11909155

[bib49] Papasimakis N, Fedotov VA, Savinov V, Raybould TA, Zheludev NI. Electromagnetic toroidal excitations in matter and free space. Nat Mater 2016; 15: 263–271. 26906961 10.1038/nmat4563

[bib50] Miroshnichenko AE, Evlyukhin AB, Yu YF, Bakker RM, Chipouline A et al. Nonradiating anapole modes in dielectric nanoparticles. Nat Commun 2015; 6: 8069. 26311109 10.1038/ncomms9069 PMC4560796

[bib51] Khorasaninejad M, Chen WT, Devlin RC, Oh J, Zhu AY et al. Metalenses at visible wavelengths: Diffraction-limited focusing and subwavelength resolution imaging. Science 2016; 352: 1190–1194. 27257251 10.1126/science.aaf6644

[bib52] Wan X, Shen XP, Luo Y, Cui TJ. Planar bifunctional luneburg‐fisheye lens made of an anisotropic metasurface. Laser Photonics Rev 2014; 8: 757–765.

[bib53] Ni XJ, Kildishev AV, Shalaev VM. Metasurface holograms for visible light. Nat Commun 2013; 4: 2807.

[bib54] Devlin RC, Ambrosio A, Wintz D, Oscurato SL, Zhu AY et al. Spin-to-orbital angular momentum conversion in dielectric metasurfaces. Opt Express 2017; 25: 377–393. 28085832 10.1364/OE.25.000377

[bib55] Cui TJ, Qi MQ, Wan X, Zhao J, Cheng Q. Coding metamaterials, digital metamaterials and programmable metamaterials. Light Sci Appl 2014; 3: e218.

[bib56] Liu S, Cui TJ, Xu Q, Bao D, Du LL et al. Anisotropic coding metamaterials and their powerful manipulation of differently polarized terahertz waves. Light Sci Appl 2016; 5: e16076. 10.1038/lsa.2016.76 PMC6059931 30167164

[bib57] Liu S, Zhang HC, Zhang L, Yang QL, Xu Q et al. Full-state controls of terahertz waves using tensor coding metasurfaces. ACS Appl Mater Interfaces 2017; 9: 21503–21514. 28580778 10.1021/acsami.7b02789

