# Supplementary material for: Giant intrinsic chiro-optical activity in planar dielectric nanostructures
Source: Light Sci Appl. 2018 Feb 23;7:17158–. doi: 10.1038/lsa.2017.158 (PMC6060067; doi:10.1038/lsa.2017.158)
Supplement: Supplementary Information [file lsa2017158x1.docx]

**Supplementary Information for:**

**Giant intrinsic chiro-optical activity in planar dielectric nanostructures**

Alexander Y. Zhu^1,†^, Wei-Ting Chen^1,†^, Aun Zaidi^1,†^, Yao-Wei Huang^1,2^, Mohammadreza Khorasaninejad^1^, Vyshakh Sanjeev^1,3^, Cheng-Wei Qiu^2^ and F. Capasso^1,*^

*^1^John A. Paulson School of Engineering and Applied Science, Harvard University, Cambridge MA 02138, United States*

*^2^Department of Electrical and Computer Engineering, National University of Singapore, 117583 Singapore, Singapore*

*^3^University of Waterloo, Waterloo Ontario N2L 3G1, Canada*

†: These authors contributed equally to this work.

*Corresponding author email: capasso@seas.harvard.edu

**S1. Magnetic dipole response**

The magnetic dipole response in high index dielectrics have been well studied in the literature^1^. It originates from vertical displacement current loops in dielectric structures with finite thickness. In order to observe a magnetic dipole resonance for a given wavelength, it is easy to see that the corresponding thickness of the structure should be $\lambda/n_{\mathrm{eff}}$, where $n_{\mathrm{eff}}$ is the effective index of the electromagnetic mode. For dipole modes $n_{\mathrm{eff}}$ is generally close to the refractive index of the material. This thickness requirement can be understood as the condition for a standing wave in the cavity formed by the structure, as shown in Fig. S1a-c. It is important to note that this magnetic dipole moment occurs in-plane, and for achiral structures it is orthogonal to the in-plane electric dipole moment.

In contrast, the electric (dipole) resonance wavelength depends primarily on the charge separation which occurs within the length of the cavity. Therefore, by tuning the aspect ratio (width : thickness) of the structure one expects to be able to tune the spectral separation between the electric dipole and magnetic dipole resonances. For cylindrical geometries a spectral overlap occurs at aspect ratio ~ 2^2,3^. This results in high transmittance in the forward-scattering direction due to the backscattered radiation from the electric and magnetic dipoles being out of phase; at a single frequency point the backscattered response will be completely canceled and the particle will only scatter forward. This is known as Kerker’s condition^4^. We reproduce this result in Fig. S1d using TiO_2_ cylinders. This physical understanding of the origin and behavior of the magnetic dipole moment in dielectrics can be used to design higher order multipoles as well.

**
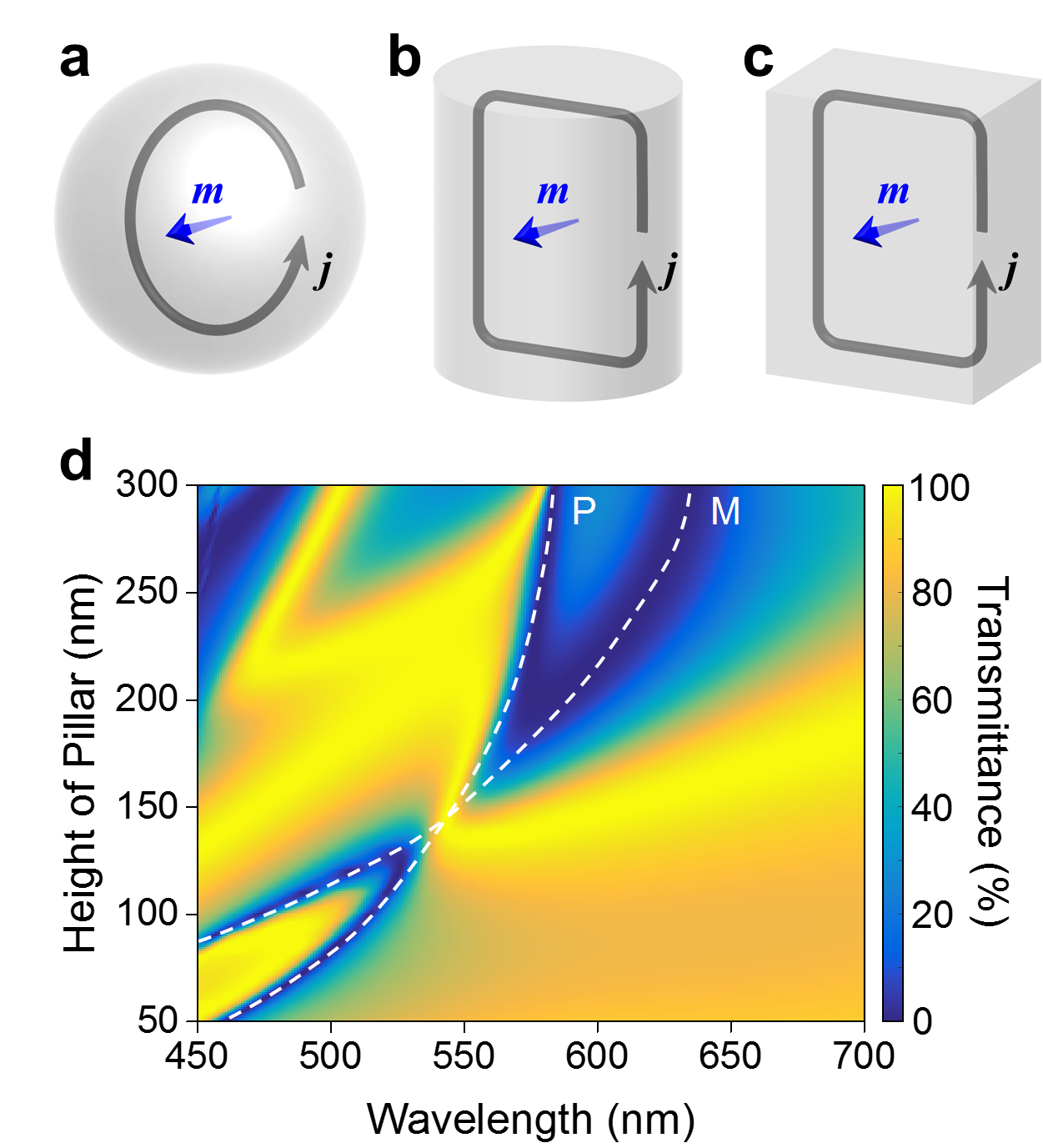
**

**Figure S1. Magnetic dipole resonance in high index dielectrics. a-c,** Schematic illustration of typical structures used to observe strong magnetic dipole resonances in high index dielectric materials, such as **(a)** a sphere, **(b)** cylinder/disk, and **(c)** a rectangular fin. In each of these cases the incident light excites a vertical displacement current loop (***j***) in the structure, which in turn generates an in-plane magnetic moment (***m***). **d,** Colormap of the transmittance of a TiO_2_ cylinder as a function of its height (fixed radius 130 nm). The spectral positions of the magnetic and electric dipole resonances can be tuned by adjusting the height and length of any given structure, i.e. their relative spectral separation is a function of the aspect ratio. There exists one frequency where the backscattered radiation from the magnetic and electric dipoles interfere destructively and completely cancel, resulting in high transmittance.

**S2. Role of waveguide layer and guided mode resonance**


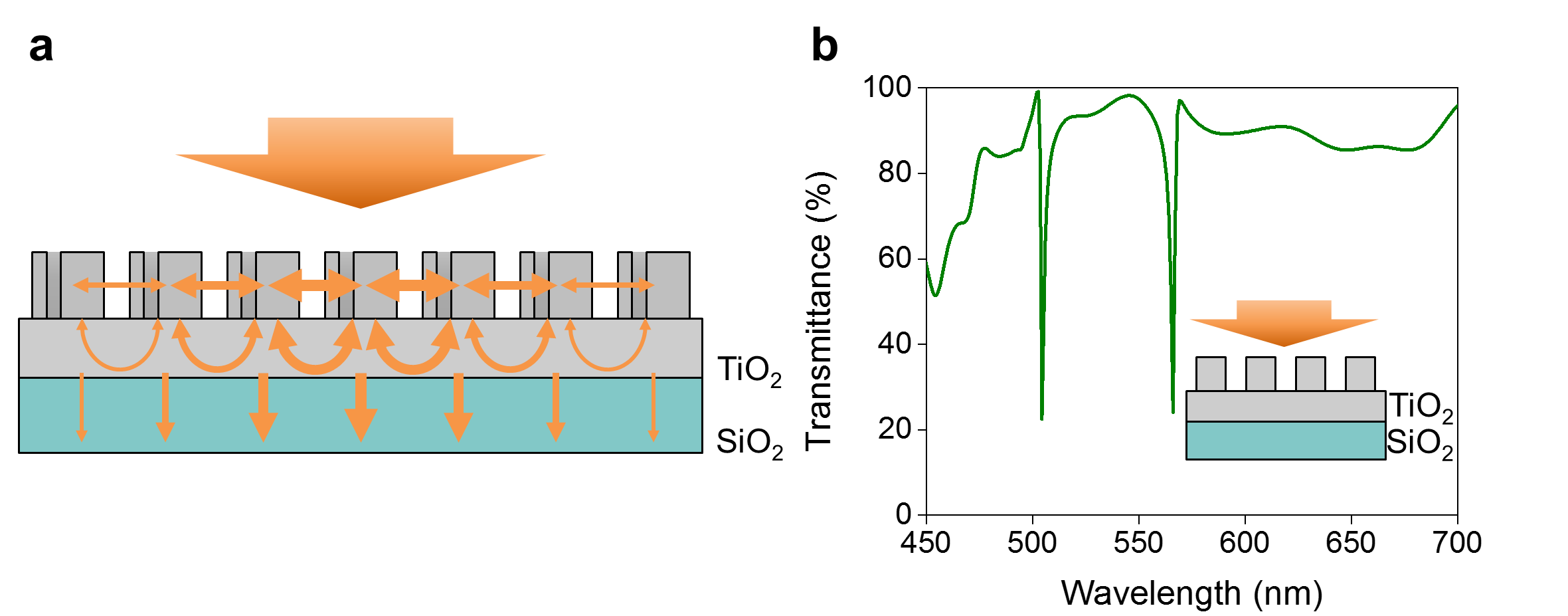


**Figure S2. Role of guided mode resonance in device design. a,** Schematic illustration (side-view) of a periodic array of gammadion nanostructures on a TiO_2_ thin film supported by a silica substrate. The period of the gammadions (500 nm) is chosen such that diffraction occurs in transmission at the operating wavelength of 540 nm. Part of the incident light is coupled into the TiO_2_ thin film which acts as a waveguide. This coupling of diffractive and waveguide modes result in guided, or leaky, mode resonances. **b,** Simulated transmittance spectrum of a TiO_2_ grating (normalized to source power), comprising infinitely long rectangles on the same TiO_2_ thin film and substrate. The widths of the stripes were chosen such that the fill factor was approximately the same as that of the gammadions. Illumination with linearly polarized light (electric field perpendicular to the long axis) reveals the presence of sharp spectral features at 510 nm and 570 nm, characteristic of guided mode resonances, as well as modulation of the transmittance envelope around 540 nm (where the transmittance ~ 100%). The presence of these spectral features result in an improved transmittance contrast. For the case of a chiral grating, light is coupled into the guided mode resonance and exhibits a transmittance dip for one helicity (here LCP) but not the other, due to their refractive index difference. In this way the use of an additional waveguide layer enhances the transmittance contrast between the two helicities and thus increases dichroism.

**S3. Optimization of structure and dimensions**


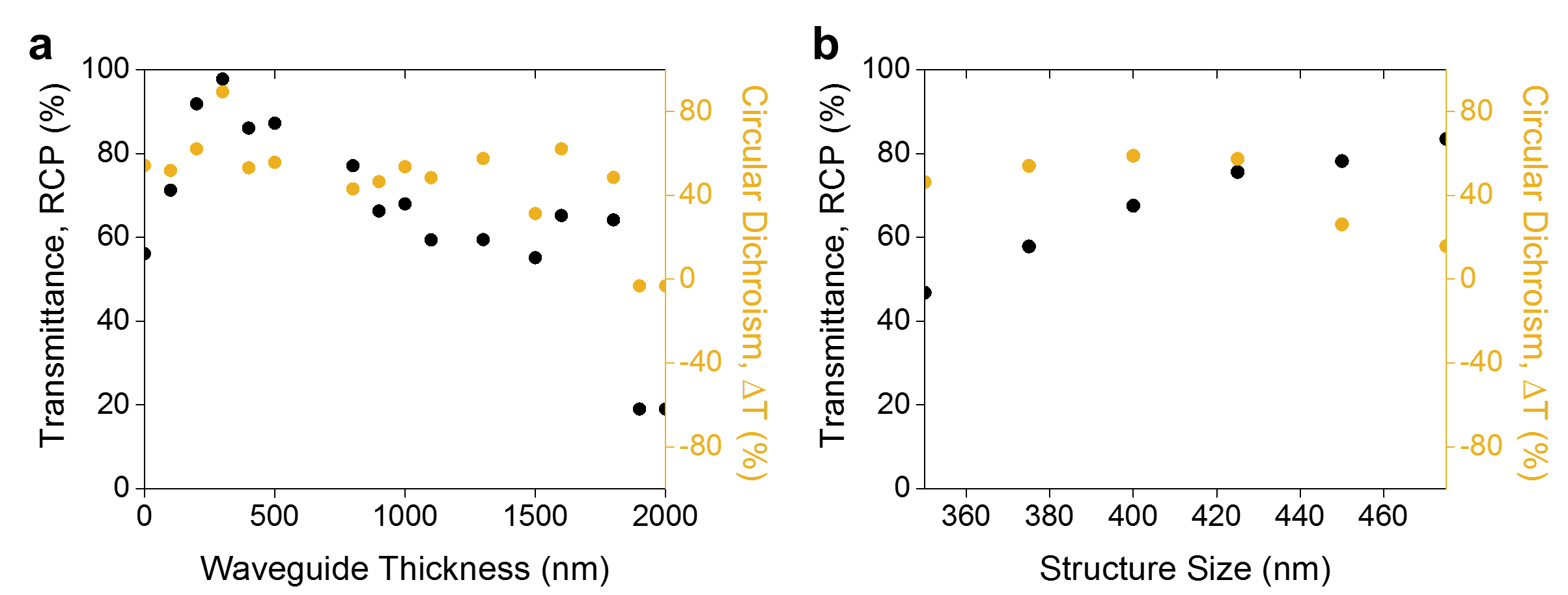


**Figure S3. Optimization of the gammadions. a,** Simulated transmittance (black) and circular dichroism (orange) achieved with our chiral structures between 500 nm – 600 nm, as a function of waveguide layer thickness. The structure achieves maximum transmittance and circular dichroism at 300 nm waveguide thickness. **b,** Optimizations of the structure as a function of its size (here the ratio of length to width is kept at 5:1) without the waveguide layer. The chiro-optical response is still evident but both transmittance and circular dichroism are weaker due to the lack of the waveguide layer which modulates the transmittance envelope.

**S4. Magnetic dipole response**


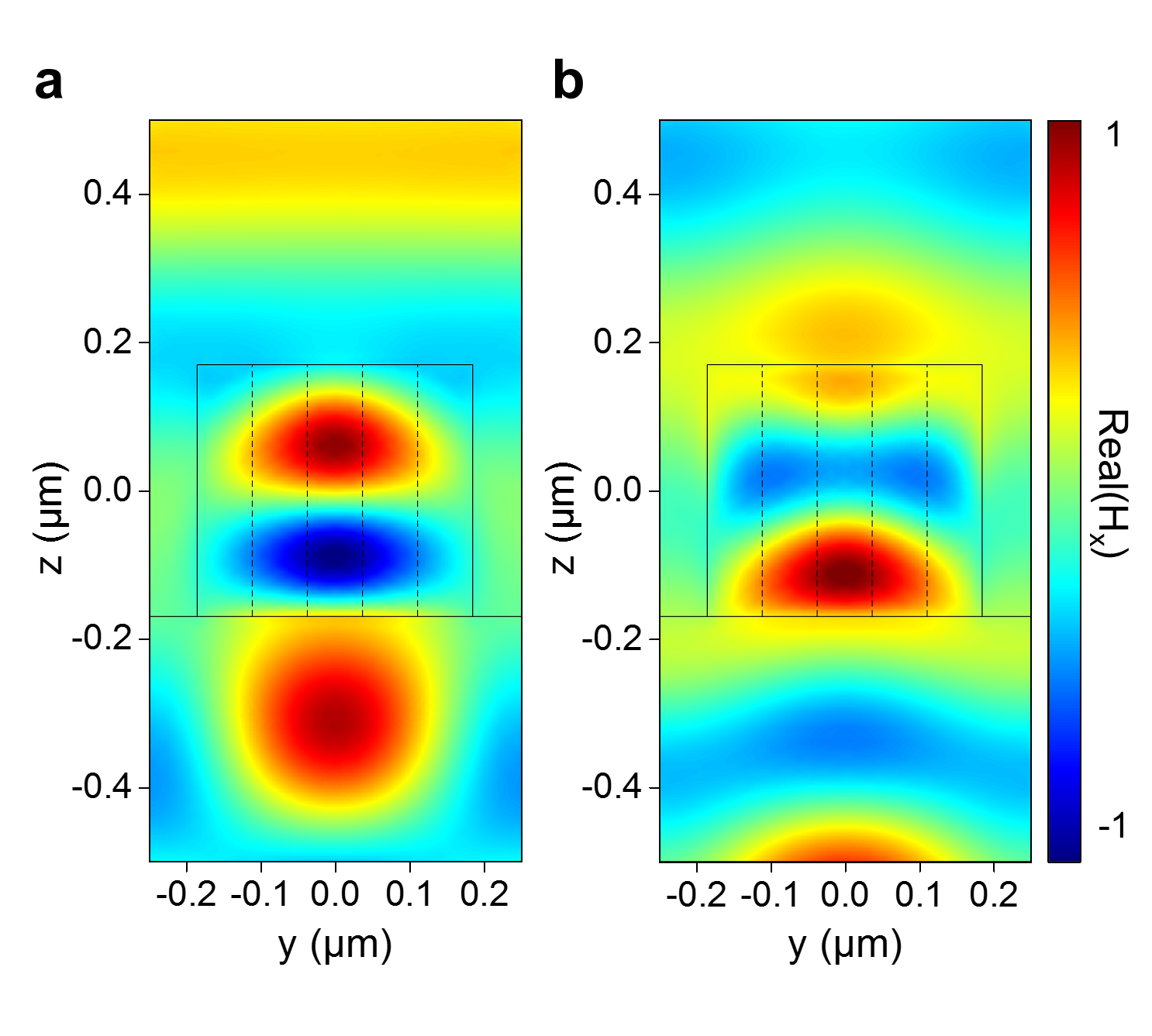


**Figure S4. Magnetic field profile (Re(H_x_)) of gammadions without the TiO2 waveguide layer.** Dominant multipole excitations can be seen to be the same as Fig. 3c and 3d in the main text, i.e. quadrupolar **(a)** and octupolar **(b)** under left and right circularly polarized incident light respectively.

**S5. Full multipole decomposition spectrum**


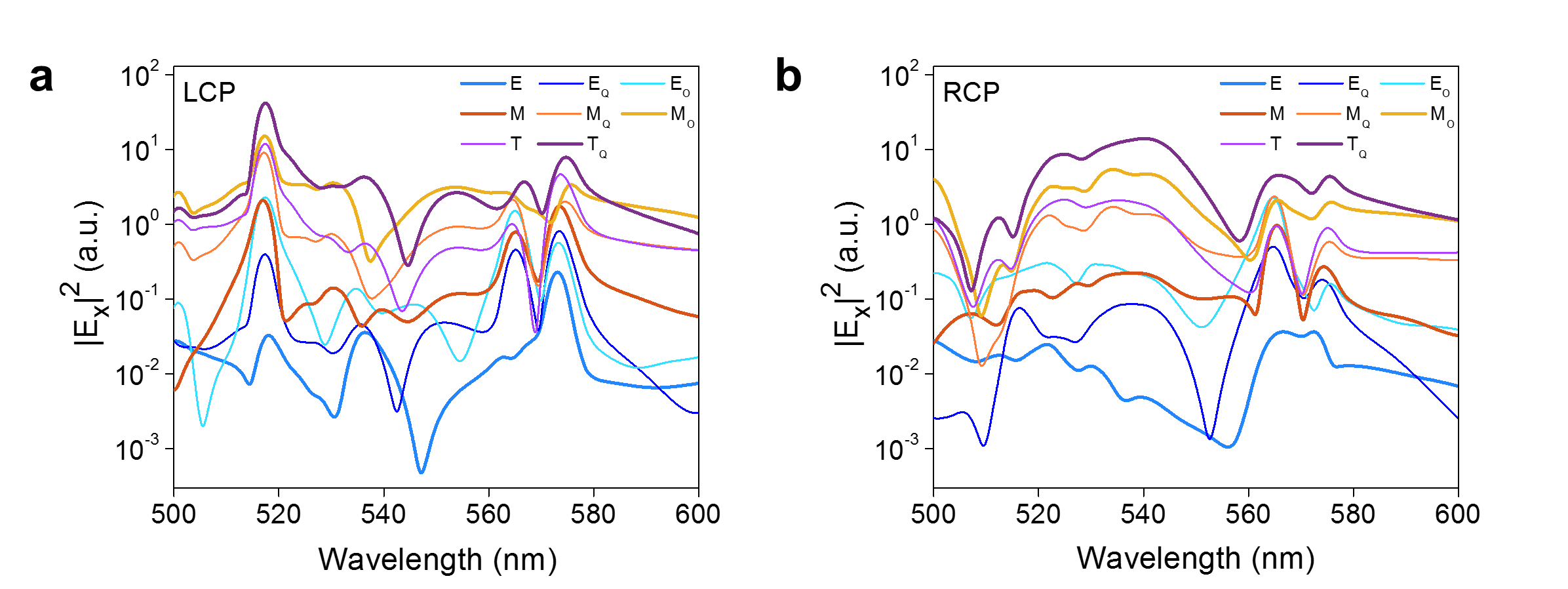


**Figure S5. Full results of multipole decomposition. a,b,** Zeroth order far-field intensity (|*E_x_*|^2^) for multipoles up to the magnetic/electric octupoles and toroidal quadrupole under **(a)** LCP and **(b)** RCP illumination. Note that the *y*-axis is in logarithmic scale.

**S6. Further characterization of chiral response**


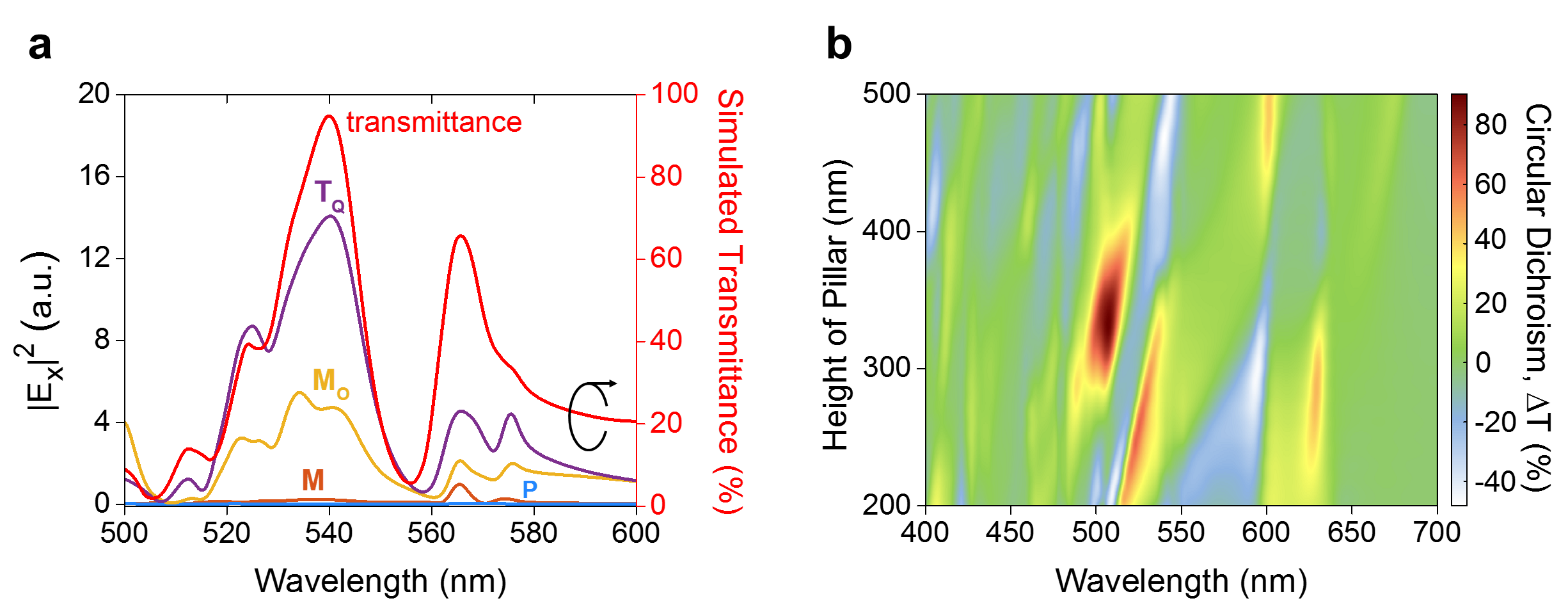


**Figure S6. Characterization of chiro-optical response. a,** The simulated 0^th^ order transmittance and individual multipole radiation spectra are overlaid. Excellent qualitative agreement is observed, indicating that the dominant multipoles in the system under RCP incidence are indeed the toroidal quadrupole (T_Q_) and magnetic octupole (M_O_). **b,** Circular dichroism map of the gammadion structures as a function of thickness and wavelength, for a fixed length and width of 370 nm and 74 nm respectively. The optimized thickness is clearly seen to be 340 nm, due to the interference between the T_Q_ and M_O_ modes.

**S7. Measurement results upon reversing direction of incident light**


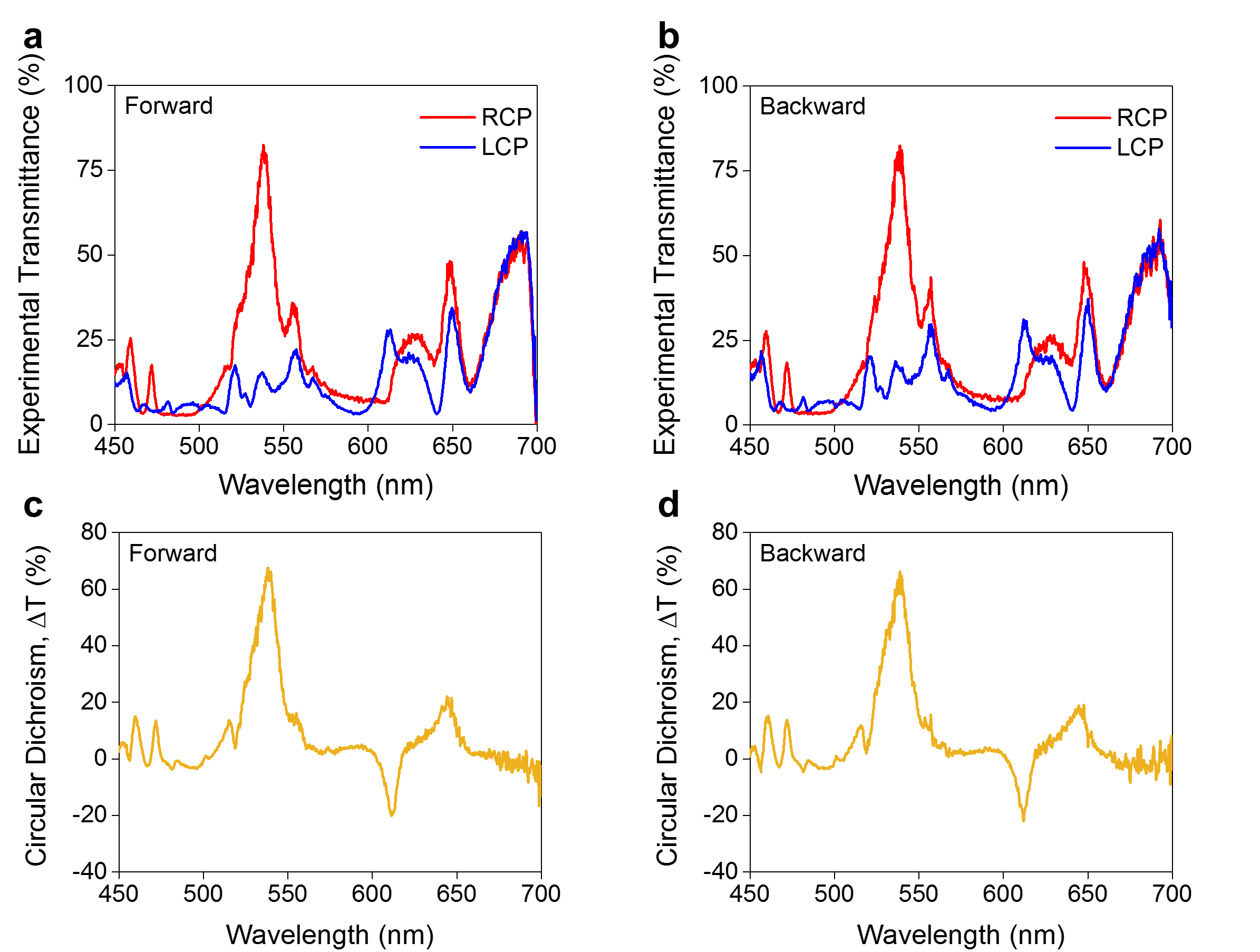


**Figure S7. Chiro-optical response upon reversing direction of incident light. a-d,** Experimental 0th order transmittance for **(a)** front and **(b)** back side incidence, as well as their circular dichroism spectra **(c), (d)** respectively. In the former case the light passes through the structure first and exits from the substrate, while in the latter the light passes through the substrate and exits from the structure. That these spectra are seen to be virtually identical is a hallmark of intrinsic chirality, here achieved with a planar structure.

**Supplementary References**

1 Kuznetsov AI, Miroshnichenko AE, Brongersma ML, Kivshar YS, Lukyanchuk B. Optically resonant dielectric nanostructures. *Science* 2016; **354**: 2472.

2 Decker M, Staude I, Falkner M, Dominguez J, Neshev DN *et al*. High-efficiency dielectric huygens’ surfaces. *Adv Opt Mater* 2015; **3**: 813-820.

3 Staude I, Miroshnichenko AE, Decker M, Fofang NT, Liu S *et al*. Tailoring directional scattering through magnetic and electric resonances in subwavelength silicon nanodisks. *ACS Nano* 2013; **7**: 7824-7832.

4 Kerker M, Wang D-S, Giles C. Electromagnetic scattering by magnetic spheres. *JOSA* 1983; **73**: 765-767.
